# Feature Point Registration Model of Farmland Surface and Its Application Based on a Monocular Camera

**DOI:** 10.3390/s20133799

**Published:** 2020-07-07

**Authors:** Yang Li, Dongyan Huang, Jiangtao Qi, Sikai Chen, Huibin Sun, Huili Liu, Honglei Jia

**Affiliations:** 1 Key Laboratory of Bionic Engineering, Ministry of Education, Jilin University, Changchun 130022, China; l_yang13@mails.jlu.edu.cn (Y.L.); huangdy@jlu.edu.cn (D.H.); sunhb18@mails.jlu.edu.cn (H.S.); liuhuili@163.com (H.L.); jiahl@jlu.edu.cn (H.J.); 2 College of Biological and Agricultural Engineering, Jilin University, Changchun 130022, China; 3 Graduate School of Agriculture, Kyoto University, Kyoto 6068502, Japan; chen.sikai.34e@st.kyoto-u.ac.jp

**Keywords:** monocular camera, farmland surface, feature point registration, attitude perception, robot vision

## Abstract

In this study, an image registration algorithm was applied to calculate the rotation angle of objects when matching images. Some commonly used image feature detection algorithms such as features from accelerated segment test (FAST), speeded up robust features (SURF) and maximally stable extremal regions (MSER) algorithms were chosen as feature extraction components. Comparing the running time and accuracy, the image registration algorithm based on SURF has better performance than the other algorithms. Accurately obtaining the roll angle is one of the key technologies to improve the positioning accuracy and operation quality of agricultural equipment. To acquire the roll angle of agriculture machinery, a roll angle acquisition model based on the image registration algorithm was built. Then, the performance of the model with a monocular camera was tested in the field. The field test showed that the average error of the rolling angle was 0.61°, while the minimum error was 0.08°. The field test indicated that the model could accurately obtain the attitude change trend of agricultural machinery when it was working in irregular farmlands. The model described in this paper could provide a foundation for agricultural equipment navigation and autonomous driving.

## 1. Introduction

With the development of computer technology, currently, smart cameras are widely used in navigation, positioning, tracking, obstacle avoidance, monitoring, etc. [1,2]. Among them, visual measurement is becoming a research hotspot, which uses a camera to capture the static single frame image or dynamic sequence images of the target [3,4]. Image processing and analysis technologies are used to measure the target structural parameters and motion parameters. It has the advantages of non-contact, fast dynamic response and high efficiency [5]. Thus, it has been widely used in the field of industrial pose measurement. Intelligent agricultural machinery equipment is one of the important technologies of modern agriculture, and its working width has gradually broadened. During field operations, the attitude angles of the wide-amplitude agricultural machine, especially the roll angle, affect the tillage depth and compressing strength of the machine, thereby impacting the tillage quality [6,7]. Therefore, the accurate roll angle of the implement could make the control of automatic navigation agricultural vehicles more precise [8], and it would help to warn of a possible roll before climbing or descending, thereby effectively assisting to decrease the loss of life and property [9]. There are many ways to obtain the roll angle of agricultural machine [10,11]. Since the camera cost is low and it can provide rich visual information, with the rapid development of hardware computing capabilities, image processing has become a popular method for acquiring the roll angle.

At present, there are many types of methods for obtaining the attitude angle of an object based on image processing [12,13]. One of them is realized by extracting the geometric features of the object. For example, Arakawa et al. implemented the attitude estimation system from the visible horizon in the images for unmanned aerial vehicles (UAVs). This system finds the horizon in the image, and then estimates attitude from the horizon. The horizon is detected using morphological smoothing, Sobel filter and Hough transform [12]. Timotheatos et al. proposed a vehicle attitude estimation method based on horizon detection, which detects the horizon line based on a canny edge and a Hough detector along with an optimization step performed by a particle swarm optimization (PSO) algorithm. The roll angle of the vehicle is determined from the angle formed by the slope of the horizon, and the pitch angle is computed using the current and initial horizon positions [14]. There is another type of method that uses the correspondence between the target model point and its imaging point to obtain the attitude angle of the object. For example, Wang presented a method for the perspective-n-point (PnP) problem for determining the position and orientation of a calibrated camera from known reference points. The method transfers the pose estimation problem into an optimal problem, which only requires solving a seventh-order polynomial and a fourth-order univariate polynomial, respectively, making the processes more easily understood and significantly improving the performance [15]. Zhang et al. proposed a method for attitude angle measurement using a single captured image to assist with the landing of small-scale fixed-wing UAVs. The method has the advantage that the attitude angles are obtained from just one image containing five coded landmarks, which reduces the time to solve the attitude angle while having more than one image in most methods [16]. These kinds of methods require the manual setting of landmarks to deal with the corresponding problems. In the actual experiment, due to the large number of target points, there is a certain error in placing the control points manually or solving the corresponding problems by computers, and solving the corresponding problems also needs large amount of computation.

In addition, the method of attitude acquisition based on SLAM (simultaneous localization and mapping) is also a research hotspot in recent years. SLAM usually refers to a robot or a moving rigid body, equipped with a specific sensor, estimating its own motion and building a model (certain kinds of description) of the surrounding environment without prior information [17]. At present, SLAM-based home service robots are widely used, and some scholars also research car autopilot and drone navigation based on SLAM [18,19]. However, few studies have been applied in complex field environments. Both this method and the visual odometer in SLAM use image feature points for registration. Considering the complex environment of the field, map building is not carried out in this paper.

Both types of methods need to extract object features, thus the feature extraction and matching methods would directly affect the applicability and accuracy of the algorithm. The efficient registration algorithm of visual images has become an important content in the study of visual image technology. In this paper, the image registration algorithm is used to obtain the rotation angle of the object, and different feature detection algorithms in image registration are compared, such as FAST corner point [20], SURF [21] and MSER [22] detection algorithms. The method in this paper overcomes the shortcomings of high demand on the shape of the object when calculating the rotation angle. There is no need for camera calibration and manual setting of marker points. The rotation angle can be obtained at the same time when matching the images.

Section 2 introduces and details our method. In Section 3, different field feature point registration models are compared. Then, based on a farmland feature point registration model, an attitude angle acquisition model for agricultural machinery is proposed. Section 4 shows the field test results and analyses. Finally, Section 5 draws the conclusions.

## 2. Theory and Method of Feature Point Registration

### 2.1. Theory of Image Registration

The core of image registration is finding a feasible mathematical model to explain the relationship between corresponding pixels in two images. Although these images may come from different angles and locations in a scene, or come from different sensors, statuses or times, it is possible to find a suitable way to register images according to different features.

Image registration includes spatial variation and grayscale transformation between two pictures. When using a two-dimensional matrix to express an image, the relationship between registration image P_i−1_ and P_i_ is

P_i_(*x*, *y*) = *g*(P_i−1_ (*f*(*x*, *y*))) (1)

where P_i−__1_(*x*, *y*) and P_i_(*x*, *y*) are the grayscale values of a pixel at (*x*, *y*), *f* is a two-dimensional space geometric transformation function and *g* is a linear grayscale transformation function.

### 2.2. Image Registration Procedure

**Figure 1:**
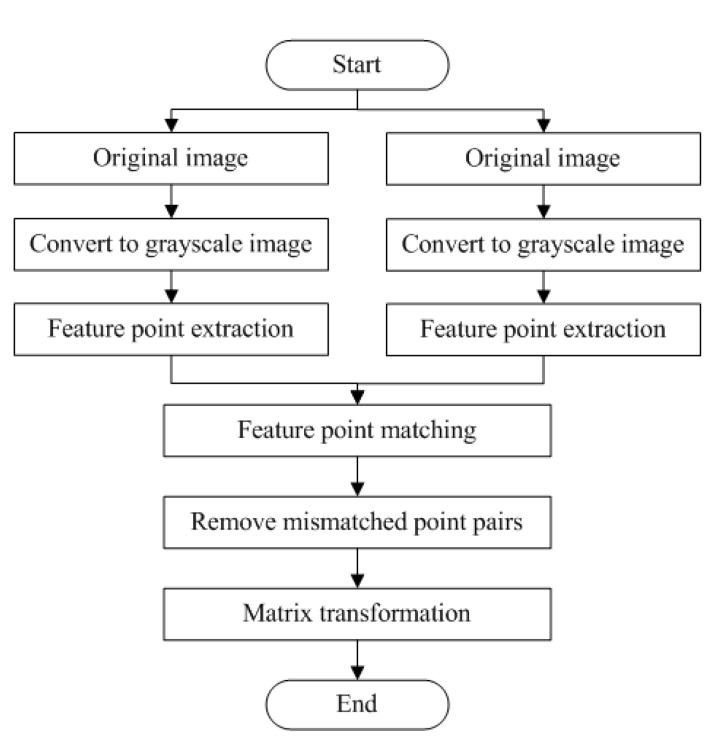
Procedure of feature-point-based image registration algorithm.

Figure 1 is the image registration algorithm procedure. Firstly, take two consecutive images P_i−1_ and P_i_. The image P_i−1_ is referred to as the original image and P_i_ is referred to as the target image. Then, convert the two images to grayscale and extract feature points from them with feature extraction algorithm, which means pinpointing the feature points on each grayscale image. Afterwards, pick out the feature descriptor (including scale and direction information) and location information from each feature point. Finally, extract local feature points from images. Next, enter the feature point registration step. Set the original image as the reference, and then match the target image with the original image, i.e. match the original image and target image with feature descriptors. After that, eliminate mismatches. Subsequently, calculate a transformation matrix with the location of feature point pairs, eventually deriving the angle of two consecutive images calculated by this matrix.

In this method, Euclidean distance is applied to measure the registration between two images. When there is a small difference in the distribution of luminance information in multiple regions of the image, a feature point in the original image may match multiple feature points in a certain region of the target image. At this time, the multiple feature points in the target image may not be correct registration points. In addition, when the feature points in the original image are known, the corresponding feature points may not be detected in the target image, and the points found directly by the closest Euclidean distance might be mismatched points. Thus, this paper uses the ratio of the closest distance to the second-closest distance to find the most obvious feature points within the set threshold, which can reduce the occurrence of the above situation. Assume that there are M1 and M2 feature points in images P_i−1_ and P_i_, respectively. For any feature point m1 in M1, the two feature points m2 and m2* with the shortest Euclidean distance from m1 in M2 correspond to distance dij and dij*. If dij ≤ *α* × dij* (in this paper, *α* is 0.6), then m1 and m2 are taken as the corresponding registration feature point pairs. This method is used to find all possible registration pairs for all the feature points in P_i−1_ and P_i_.

### 2.3. Method of Image Registration

The feature-based image registration algorithm mainly includes feature extraction, feature description and feature registration. If the ratio of correctly matched feature pairs is greater than a certain proportion, M-estimator Sample Consensus (MSAC) algorithm [22] is applied to obtain these pairs, and then parameters of geometric transformation model between two images are calculated. Thus, before executing image registration, the priority is extracting features.

Feature extraction mainly includes feature detection and feature description, and feature detection algorithms consist of two classes: one is a feature-point-based detection algorithm and the other is a feature-area-based detection algorithm. Corner point detection is a commonly used feature point detection algorithm, such as Harris corner point, SUSAN corner point, FAST corner point, SURF and scale-invariant feature transform (SIFT) descriptor [23]. FAST considers 16 pixels in a cirle near the pixel point. For example, p is the center pixel point; if the value of n consecutive points from 16 pixel points in the circle are greater or smaller than the value of the center pixel p, then the center point will be the corner point. SURF is an improved and accelerated version of SIFT. SIFT detects extreme points in scale space to find feature points in different scale space and calculate the direction of key points. SURF operates on the integral image. The value of each pixel in the integral image is the sum of all elements in the upper left corner of the corresponding position on the original image; thus, acceleration can be realized. Because the FAST corner detection algorithm is fast and SURF algorithm is faster than SIFT algorithm while maintaining high accuracy, these two algorithms were chosen as representatives for comparison.

Feature-area-based detection methods find feature points and their surrounding area, which contains feature points and other information [24]. The MSER method denotes a set of distinguished regions that are detected in a grayscale image. It shows better adaptation on detecting gray consistency regions with strong discriminative boundary and structured and textured images. MSER also has better performance when light intensity changes compared to other area detection operators [25].

The image registration algorithm based on FAST corner point (hereafter, FAST−M), SURF (hereafter, SURF−M) and MSER (hereafter, MSER−M) was selected to build the image registration algorithm. For the sake of choosing the most suitable algorithm to build field feature point image registration model, the images of two typical field operations were tested in MATLAB. The software for the experiment was windows 10, MATLAB R2014a and Microsoft visual C++ 2015; the processor was Intel Core i7-8700 CPU, running at 3.2 GHz, 16.0 GB RAM.

## 3. Field Feature Point Registration Model and Application

### 3.1. Comparation and Analyze of Detection Operator

**Figure 2:**
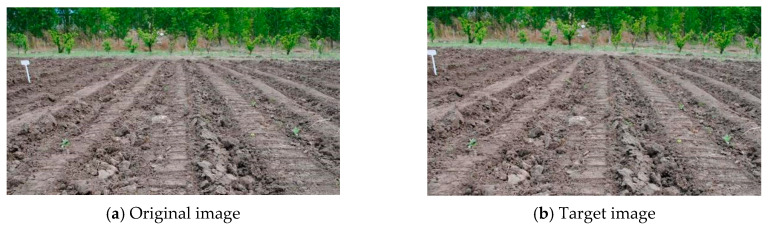
Original images and target images for scale test.

Considering the working environment for the agricultural machinery, images captured by a camera in the field may be affected by scaling, noise, brightness, etc. Therefore, the effects of image scaling, image noise and image brightness on the registration accuracy were tested, respectively. According to the results of the communique of the second national land survey data main achievements [26], the area of land whose slope is less than 2° is 57.1%. Therefore, the actual deflection angle between the two original images and the target image was set as 2° in the experiment. The images used for the three tests are displayed in Figure 2. To test the effects of image scaling, image noise and image brightness on the registration accuracy, firstly, the target image was obtained by rotating and cropping the original image. Then, three different operations were performed on the image shown in Figure 2b to obtain new target images. Finally, they were registered with the original images, respectively. Each test was run 100 times, and the average value of the time required for program operation and the angle error were taken as the evaluation indices.

#### 3.1.1. Experiment on the Effect of Scaling on Registration Accuracy

**Figure 3:**
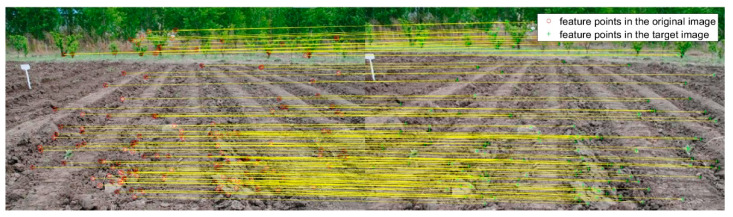
The registration result of FAST−M for target image in Figure 2b.

**Figure 4:**
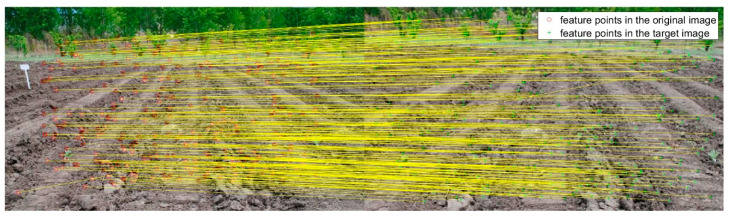
The registration result of SURF−M for target image in Figure 2b.

**Figure 5:**
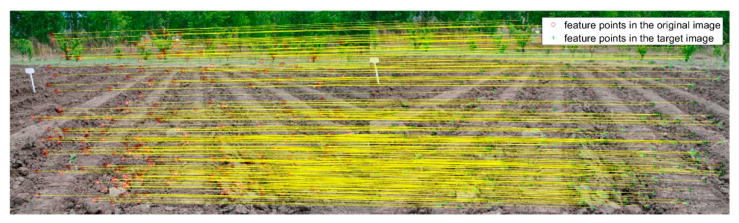
The registration result of MSER−M for target image in Figure 2b.

Target images were not affected by brightness and noise relative to the original image. Based on the target image in Figure 2b, different scale (0.6, 0.8, 1.2 and 1.4) transformations using the resize function in OpenCV were performed, and then the transformed images were used for experiments. The test results for target image in Figure 2b are shown in Figure 3, Figure 4 and Figure 5, respectively.

**Figure 6:**
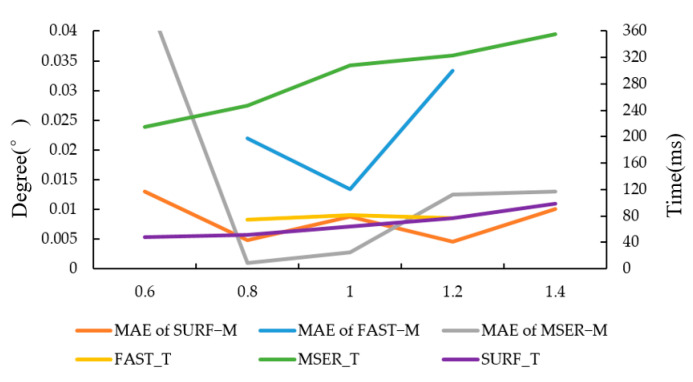
The result of FAST−M, SURF−M and MSER−M. MAE means mean absolute error, while FAST_T, MSER_T and SURF_T mean the running times of the algorithms, respectively.

The experimental results of FAST−M, SURF−M and MSER−M are shown in Figure 6. According to the curves in Figure 6, the scale change has a certain influence on the accuracy of MSERF−M and FAST−M algorithms, and it has the greatest influence on the accuracy of Fast−M algorithm. When the transform scale is 0.6 or 1.4, Fast−M algorithm cannot complete the registration. When the accuracies of the SURF−M and MSERF−M algorithms are close, the SURF−M algorithm takes less time than the MSERF−M algorithm. It can be seen that SURF−M algorithm is the best choice at different scales.

#### 3.1.2. Experiment on the Effect of Noise on Registration Accuracy

**Figure 7:**
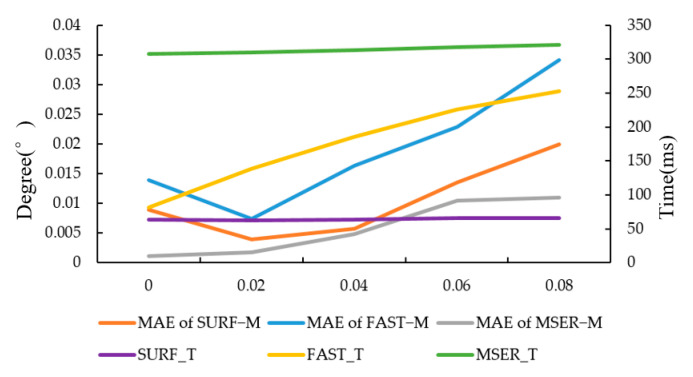
The result of FAST−M, SURF−M and MSER−M. MAE means mean absolute error, while FAST_T, MSER_T and SURF_T mean the running times of the algorithms, respectively.

Target images were not affected by brightness relative to the original image. To verify the adaptability of the algorithms to noise variation, salt and pepper noise with noise densities of 0.02, 0.04, 0.06 and 0.08 were added to the target image in Figure 2b, respectively, and then registered with the original image. The result of FAST−M, SURF−M and MSER−M are shown in Figure 7.

As shown in Figure 7, with the noise intensity increasing, the accuracy of the three algorithms all decreases, and the accuracy of the FAST−M algorithm decreases the most. In terms of the running time, with the increase of noise intensity, the time of FAST−M algorithm increases more, while the times of SURF−M and MSERF−M algorithm increase less. The reason is that the FAST corner detection algorithm is more sensitive to noise. When the noise is strengthened, it detects too many noise points, thereby increasing the running time, and the accuracy is reduced accordingly.

#### 3.1.3. Experiment on the Effect of Brightness on Registration Accuracy

To verify the adaptability of the algorithms to brightness variation, target images were affected by brightness relative to the original image. The brightness of target images in Figure 2b was changed with MATLAB and then registered with the original images. The specific operation of brightness change means increasing or decreasing by a certain value each pixel in the image. In this study, four values were selected: −50, −30, 30 and 50.

**Figure 8:**
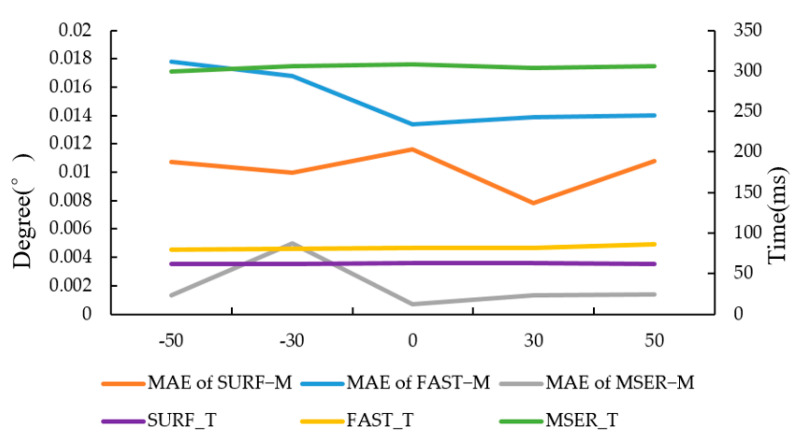
The result of FAST−M, SURF−M and MSER−M. MAE means mean absolute error, while FAST_T, MSER_T and SURF_T mean the running times of the algorithms, respectively.

The results of detection operators FAST−M, SURF−M and MSER−M are shown in Figure 8. As shown in Figure 8, the change of image brightness has a certain impact on the accuracy of the three algorithms but has little effect on running time. Overall, the SURF−M algorithm takes the least time. Although the MSERF−M has the highest accuracy, it also takes the most time.

#### 3.1.4. Conclusion and Discussion

With regard to running time, MSER−M was the slowest and FAST−M was the fastest. In terms of accuracy, all three algorithms had error less than 0.1°. FAST−M algorithm was more sensitive to noise and scale transformations than the other algorithms. Although the accuracy of MSER−M was the highest for most cases, the running time was the longest. Considering the perspective of running time and accuracy, the best registration algorithm was SURF−M. As a result, in this study, SURF−M was elected algorithm to register feature points on images.

### 3.2. Farmland Feature Point Registration Model and Procedure

#### 3.2.1. Farmland Feature Point Registration Model

When an agricultural machinery is working in a straight line in the field, the environment in front of or behind the machinery changes slightly within a short time interval. To eliminate the influence caused by the variation in depth, in the actual field test, the program collected pictures within a short interval. Because the agricultural machinery works along a straight line at low speed, compared with the field environment, the depth change in this short period of time would not have a significant impact on parallax. In the experiment, a monocular camera was mounted to agricultural machinery for recording field images. Field images were captured in real-time when agricultural machinery was working. Then, field images were registered by using the SURF−M algorithm, and the rotation angle was calculated from the transformation matrix of the two consecutive images.

**Figure 9:**
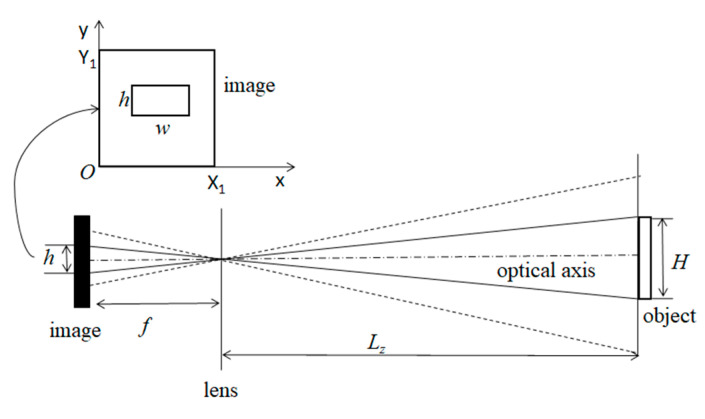
The 2D imaging model of a camera.

In Figure 9, *f* is the focal length; L_z_ is the distance between object and lens; X_1_ and Y_1_ are the height and width of the image plane, respectively; H is the actual size of object; and h and w are height and width of objects in camera image plane coordinate system, respectively.

(2) $$\begin{array}{cc} \mathrm{Referring}\;\mathrm{to}\;\mathrm{the}\;\mathrm{triangle}\;\mathrm{similarity}\;\mathrm{principle}: & \\ & \frac{H}{h}=\frac{L_{z}}{f} \end{array}$$

That is, the object in reality is proportional to the object in the image. When the camera rotates around the optical axis, the object in the image plane also rotates. Because the rotation angle of the object in image plan equals the camera rotation angle, the rotation angle between two consecutive images can be derived, and then the camera rotation angle can be obtained. When the camera is fixed on the agricultural machinery, the corresponding axes of the two coordinate systems are parallel in the direction of the optical axis of the camera. Thus, the rotation angle of the camera is the roll angle of the agricultural machinery.

The process of finding the roll angle by the transformation matrix is as follows. After the matching pairs from images are obtained, the transformation relationship between images can be estimated. During the field test, the camera was facing down and at an angle of 15° to the horizontal; thus, most of the area in the captured image was the field. At this time, the feature points on the field were approximately on the same plane, thus the homography matrix was used to calculate rotation matrix. Suppose that, for the coordinates A($x_{1}$, $y_{1}$) of any point on the original map, after the transformation of the homography matrix H, the corresponding coordinate points A’($x_{2}$, $y_{2}$) can be found on the target image; then, the transformation formula is as follows:

(3) $$\left(\begin{array}{c} x_{2}\\ y_{2}\\ 1 \end{array}\right)=H\left(\begin{array}{c} x_{1}\\ y_{1}\\ 1 \end{array}\right)=\left(\begin{array}{ccc} h_{11} & h_{12} & h_{13}\\ h_{21} & h_{22} & h_{23}\\ h_{31} & h_{32} & h_{33} \end{array}\right)\left(\begin{array}{c} x_{1}\\ y_{1}\\ 1 \end{array}\right)$$

Then, we can get:

(4) $$x_{2}=\frac{x_{1}h_{11}+y_{1}h_{12}+h_{13}}{x_{1}h_{31}+y_{1}h_{32}+h_{33}}$$

(5) $$y_{2}=\frac{x_{1}h_{21}+y_{1}h_{22}+h_{23}}{x_{1}h_{31}+y_{1}h_{32}+h_{33}}$$

It could be written as:

(6) $$x_{1}h_{31}x_{2}+y_{1}h_{32}x_{2}+h_{33}x_{2}-x_{1}h_{11}-y_{1}h_{12}-h_{13}=0$$

(7) $$x_{1}h_{31}y_{2}+y_{1}h_{32}y_{2}+h_{33}y_{2\;}-x_{1}h_{21}-y_{1}h_{22}-h_{23}=0$$

Then, we can get:

(8) $$\left(\begin{array}{ccccccccc} -x_{1} & -y_{1} & -1 & 0 & 0 & 0 & x_{1}x_{2} & y_{1}x_{2} & x_{2}\\ 0 & 0 & 0 & -x_{1} & -y_{1} & -1 & x_{1}y_{2} & y_{1}y_{2} & y_{2} \end{array}\right)h=0$$

The homography matrix H is multiplied by the number a. Because the new homography matrix turns the homogeneous point A into the homogeneous point aA, and the points on the image corresponding to aA and A are the same, then aH and H have the same effect. Therefore, there are only eight unknowns in the homography matrix H. Generally, in actual processing, it is usually multiplied by a non-zero factor so that *h*_33_ = 1. H contains eight unknowns and requires eight equations to solve, which can be solved through four sets of corresponding points. After obtaining the homography matrix, it is decomposed into the corresponding rotation matrix according to the decomposition method [27]. Finally, the roll angle can be obtained based on the rotation matrix.

#### 3.2.2. Procedure of Farmland Feature Point Registration

**Figure 10:**
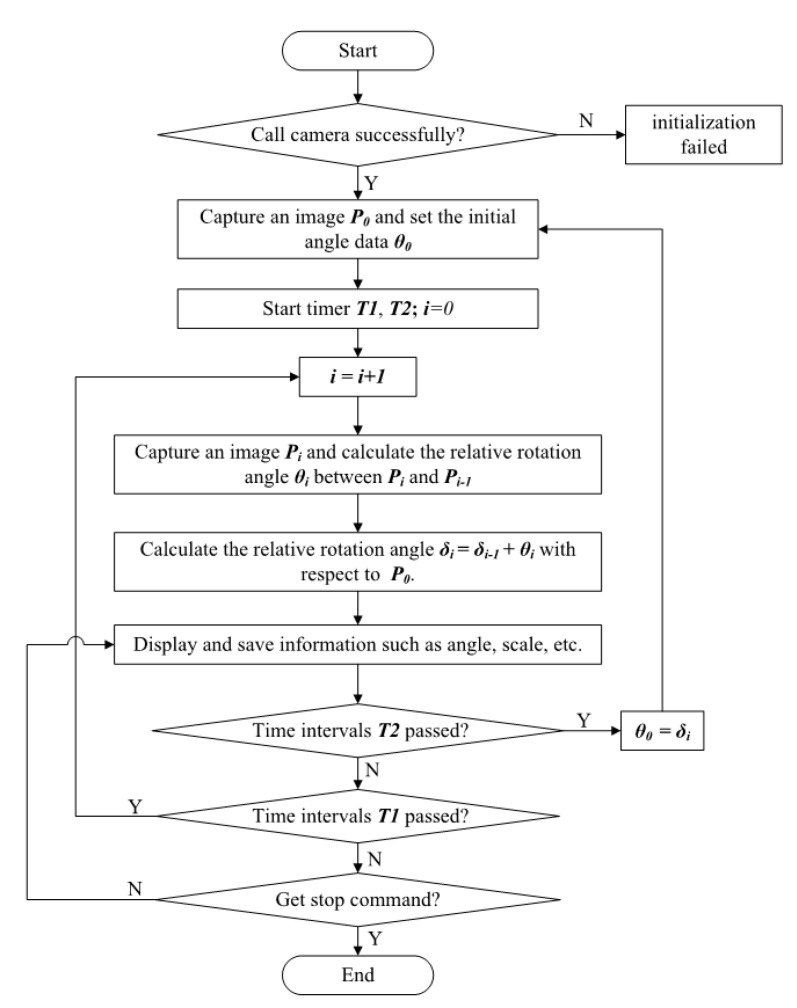
Work flow chart of the system.

The workflow of the attitude angle acquisition model is displayed in Figure 10. First, load an initial field image *P*_0_ as the original image at the initial state of operation, choose *θ*_0_ as the initial angle, and set the initial image *P*_0_ as the original image in comparison with images received later. After that, capture an image every certain period of time T1 (T1 = 1000 ms) and calculate the rotation angle *θ_i_*, between images P_i−1_ and P_i_. Then, calculate the relative rotation angle *δ_i_ = δ_i_−_1_ + θ_i_* of the loaded image with respect to the original image. This is the description of the first loop.

To eliminate the effect brought by distance variation, a new loop is needed. Before the new loop starts, when there is a certain time interval T2 from the initial time, capture a new initial image P_0′_ as the original image and angle data *θ*_0′_ (*θ*_0′_ is the relative angle *δ_i_* of the last image P_i_ in the previous loop). Choose the original image as the template in comparison with images received later. After that, the new loop starts. In the new loop, load the image every certain period of time T1 (T1 = 1000 ms) and calculate the rotation angle *θ_i_* between images P_i−1_ and P_i_. Then, calculate the relative rotation angle *δ_i_ = δ_i−1_ + θ_i_* of the loaded image with respect to the original image. This is the description of the second loop.

The hardware required by the system includes a monocular USB camera and a computer, and the running software is MATLAB R2014a. The attitude angle acquisition model of agricultural machinery is constructed by using SURF−M algorithm, and the system is built in MATLAB GUI.

## 4. Field Test and Analyses

To testify the feasibility of the attitude acquisition system, a field test was conducted in Boli county, Heilongjiang Province, China.

Experimental devices included the 2BGD-6 soybean variable fertilization seeder, an 88.2-kw CASE IH PUMA 210 tractor, a monocular camera, a digital level ruler (CHERVON Trading Co., Ltd., Nanjing, China, DEVON 9409.1) and the monitoring terminal with attitude angle acquisition model. The display accuracy of the digital level ruler is ±0.05°.

**Figure 11:**
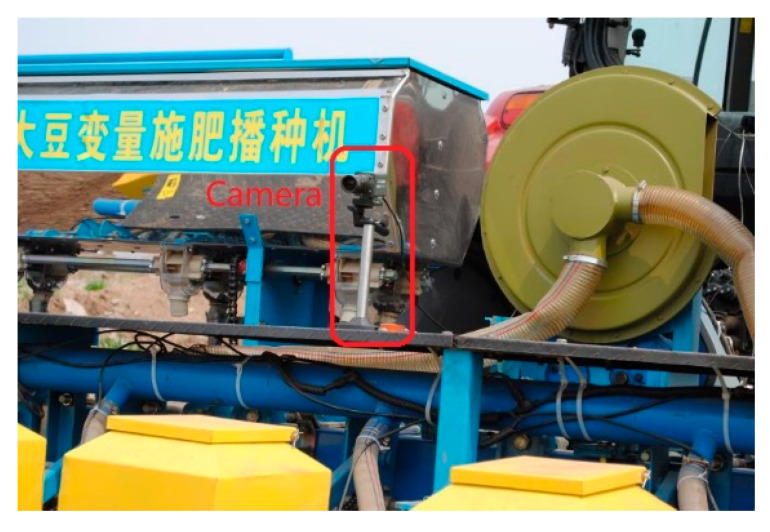
Test field.

**Figure 12:**
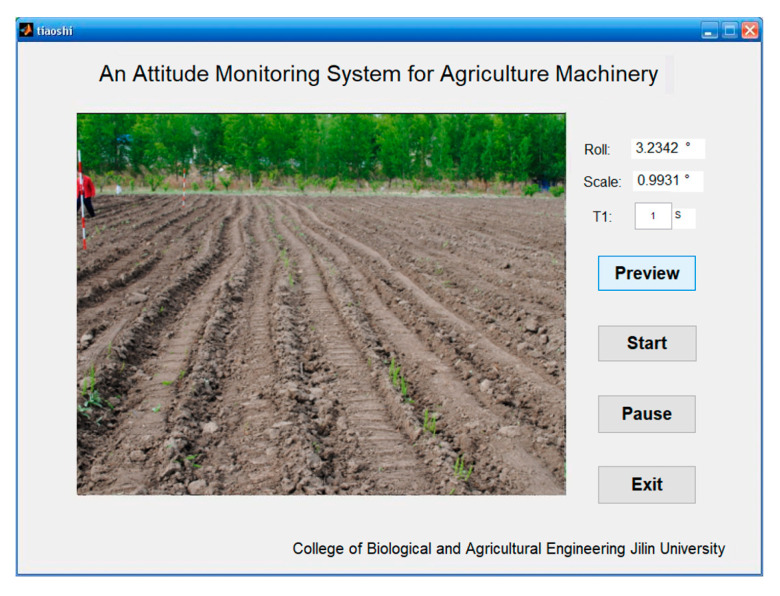
Program operation interface.

During the test, the facilities were installed as shown in Figure 11. The camera was facing down and at an angle of 15° to the horizontal; thus, most of the area in the captured image was the field. In the dynamic comparison test, the speed of the tractor was 3.6 km/h. The tractor operated along a straight line. When the speed of the tractor was stable, the monitoring test started. The frequency of attitude monitoring was 1 Hz, and a marking point was made every 1 m along the ridge with a tape measure along the driving direction of the seeder. At each marking point, the angle of the agricultural machine was collected as a true angle value by a digital angle ruler. At the same time, the image was collected through the monitoring terminal at each marking point, and the measured value was displayed and saved in real-time. The interface of the monitoring system is shown in Figure 12. By comparing the two values, the performance of the image feature point registration algorithm, which was applied to the roll angle measurement, was evaluated.

**Figure 13:**
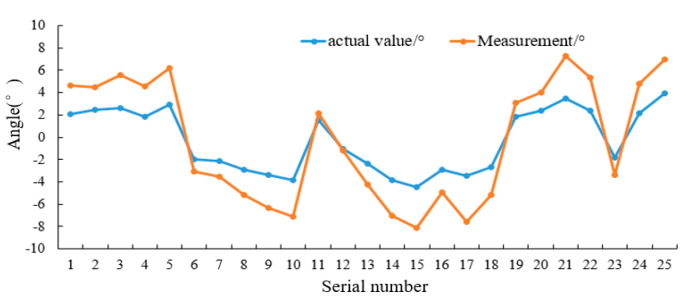
The field test result.

As displayed in Table 1 and Figure 13, comparing the angle value calculated by the attitude monitoring algorithm with the angle value measured by the digital display angle ruler, the maximum, minimum and average values of the absolute error of the attitude monitoring algorithm were 0.97°, 0.08°, and 0.61°, respectively.

The test results reveal that, when the agricultural machinery was working in the field, if its attitude changed, the model established could accurately perceive the attitude change trend and obtain the rotation angle of the agricultural machinery. However, there was a certain error in the attitude angle obtained by the system. The cause of the error is mainly because the camera was installed on the agricultural machine. The vibration of the agricultural machinery during the operation had an impact on the camera, which caused a slight deviation of the object in the captured images, resulting in a certain error in the rotation angle. In this study, since the feature variation between two images was small in a short time, the rotation angle of the two images could be obtained, and then the rotation angle of the camera at different times could be obtained. After obtaining the rotation angle of the camera, the roll angle of the agricultural machinery could be acquired. Compared with the SLAM algorithm, the function of the algorithm in this paper is relatively simple, but the algorithm is more concise, and the complexity is lower.

## 5. Conclusions

This paper presents a method for obtaining feature points with a monocular camera. Image registrations algorithm based on common image feature detection algorithms, such as FAST, SURF and MSER feature detection algorithm, were studied. By comparing the running time and accuracy of the three detection algorithms, the results demonstrate that FAST−M algorithm was more sensitive to noise and scale transformation than the other algorithms. Regarding the running time, the MSER−M algorithm took the longest time, followed by SURF−M, while the FAST−M algorithm took the shortest time. With respect to accuracy, the errors of the three algorithms are all less than 0.1°. In light of the test results, the SURF−M algorithm was selected for field feature point registration.

In this study, model for obtaining the attitude angle based on a monocular camera was built. When tested in the field, the average error of the rolling angle was 0.61°, and the minimum error was 0.08°. Field experiments indicated that the model could accurately obtain the attitude change trend of agricultural machinery. Due to the vibration of the machine, there was a certain error in the attitude angle obtained by the system. In the future, how to eliminate the influence of vibration on the accuracy of attitude angle acquisition should be deeply studied.

## Figures and Tables

**Table 1:** The field test result.

Serial Number	Actual Value/°	Measurement/°	Absolute Error/°
1	2.05	2.56	0.51
2	2.44	2.03	0.41
3	2.58	2.96	0.38
4	1.86	2.67	0.81
5	2.89	3.32	0.43
6	−1.98	−1.07	0.91
7	−2.17	−1.34	0.83
8	−2.89	−2.26	0.63
9	−3.36	−2.98	0.38
10	−3.86	−3.24	0.62
11	1.54	0.57	0.97
12	−1.05	−0.18	0.87
13	−2.35	−1.86	0.49
14	−3.81	−3.23	0.58
15	−4.45	−3.67	0.78
16	−2.89	−2.06	0.83
17	−3.45	−4.11	0.66
18	−2.64	−2.56	0.08
19	1.86	1.23	0.63
20	2.35	1.67	0.68
21	3.45	3.86	0.62
22	2.36	2.98	0.41
23	−1.84	−1.56	0.28
24	2.17	2.64	0.47
25	3.94	3.06	0.88
Maximum Error	——	——	0.97
Minimum Error	——	——	0.08
Average Error			0.61
